# Sigmoid Volvulus as One of the Differential Diagnoses for Bradycardia and Hypotension: A Case Report

**DOI:** 10.7759/cureus.107375

**Published:** 2026-04-20

**Authors:** Hiromu Suenaga, Ryunosuke Hashikawa, Masakatsu Usui, Kenji Miki

**Affiliations:** 1 Emergency Department, Uji Tokushukai Medical Center, Kyoto, JPN

**Keywords:** bezold-jarisch reflex, bradycardia, colonic distension, emergency endoscopy, shock, sigmoid volvulus, vagus nerve

## Abstract

Bradycardia and hypotension are well-recognized clinical presentations in the emergency department, and common causes include cardiac, metabolic, or drug-related factors. However, gastrointestinal pathologies are seldom considered within the initial differential diagnosis. We report the case of a 75-year-old man who presented with light-headedness due to life-threatening bradycardia and hypotension. A chest radiograph incidentally revealed marked colonic distension beneath the diaphragm. After stabilizing his vital signs with vasopressors, a computed tomography revealed a sigmoid volvulus. Emergency colonoscopic decompression led in a rapid stabilization of his vital signs, permitting discontinuation of vasopressor support. This case emphasizes that gastrointestinal disorders, like sigmoid volvulus, should be considered in the initial differential diagnosis of bradycardia and hypotension.

## Introduction

Bradycardia and hypotension are common presentations in the emergency department (ED). These presentations can sometimes be life-threatening and require an immediate differential diagnosis, with an approach tailored to the underlying cause. Differential diagnoses include cardiogenic, metabolic, or drug-related causes [1,2]. However, gastrointestinal disorders are seldom considered as a primary cause of bradycardia and hypotension [3]. Here, we describe a case of sigmoid volvulus presenting with bradycardia and hypotension, which rapidly resolved following endoscopic decompression.

## Case presentation

A 75-year-old man was transferred to our ED immediately after the onset of acute light-headedness. He had been functionally independent and ambulatory prior to presentation. He denied experiencing chest or abdominal pain, dyspnea, or loss of consciousness. He also did not complain of any digestive symptoms such as nausea, vomiting, or constipation, but his abdomen was distended. His past medical history included congestive heart failure and angina pectoris, treated with percutaneous coronary intervention to the left circumflex artery. His regular medications included clopidogrel 25 mg, azosemide 30 mg, bisoprolol 2.5 mg, and empagliflozin 10 mg once daily.

**Figure 1:**
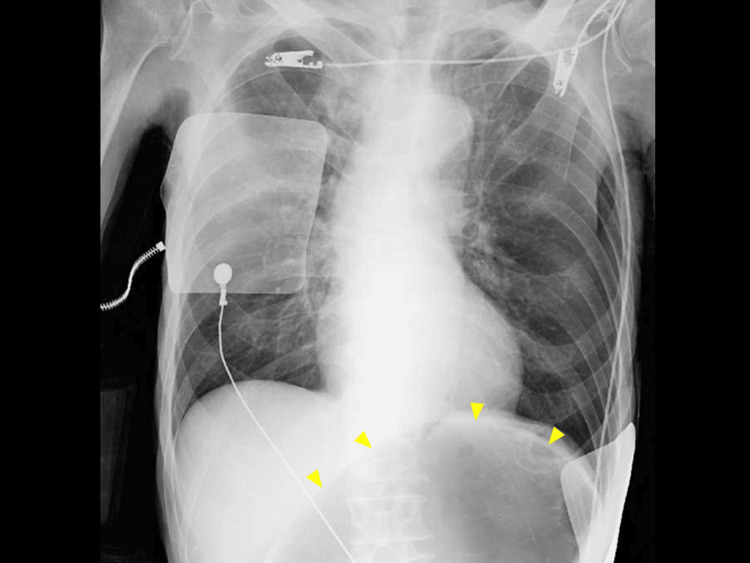
Chest radiograph It showed no chest lesions, but a colon distension beneath the diaphragm.

Upon admission to the ED, his vital signs were unstable, and as follows: blood pressure (BP; normal range: approximately 90-120/60-80 mmHg), respiratory rate 17 breaths/min (normal range: 12-20 breaths/min), oxygen saturation 98% on room air (normal value: approximately ≥95% on room air), body temperature 36.4℃ (normal range: 36.0-37.5℃), heart rate (HR) 40-50/min (normal range: 60-100/min), with a regular rhythm. His Glasgow Coma Scale score was 14 (E3V5M6), indicating mild alteration of mental status. Physical examination revealed slightly cold extremities, no jugular vein distension, and normal breath sounds. Blood gas test demonstrated mild lactic acidosis (lactate level of 26 mg/dL; normal value: ≤18 mg/dL) without electrolyte abnormalities. The electrocardiogram showed sinus rhythm without obvious ST-segment elevations or PR and QT interval prolongation. Point-of-care echocardiogram revealed no regional wall motion abnormalities. A chest radiograph showed no apparent pulmonary lesions but unexpectedly marked colonic distension beneath the diaphragm (Figure 1).

**Figure 2:**
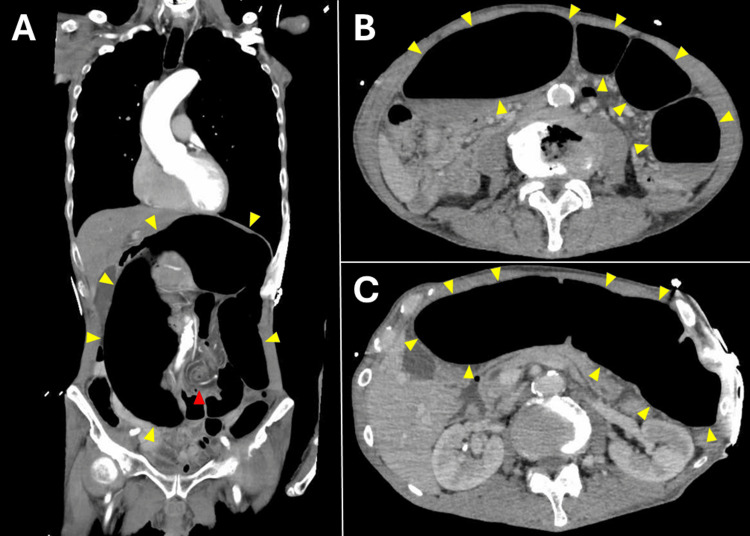
CT scan (A) Coronal view; (B, C) Axial view. A whirl sign (red arrow head) and distended colon (yellow arrow heads) were consistent with sigmoid volvulus.

Despite aggressive fluid resuscitation, his BP remained unstable. Continuous infusion of noradrenaline at 0.15 µg/kg/min increased BP to 94/57 mmHg with his HR of 45-60/min. Noradrenaline was administered for approximately one hour prior to the colonoscopic decompression, with the dose adjusted as needed to maintain BP. An abdominal computed tomography (CT) revealed a markedly distended and twisted colon, consistent with sigmoid volvulus (Figure 2).

Emergency colonoscopic decompression was performed, after which his BP and HR were gradually stabilized, allowing tapering of the noradrenaline infusion. At the end, his BP was 129/61 mmHg and HR was 65/min without vasopressor support. He was admitted for further examination and management. We suspended bisoprolol to prevent a recurrence of symptoms just in case. Finally, he was discharged on hospital day five without recurrence of symptoms.

## Discussion

The patient presented with light-headedness accompanied by marked bradycardia and hypotension. An initial chest radiograph incidentally revealed colonic distension beneath the diaphragm. After stabilization of hemodynamics with vasopressor support, CT demonstrated a sigmoid volvulus. The volvulus was resolved by endoscopic decompression, and his vital signs rapidly recovered.

Colonic distension can influence cardiovascular regulation by stimulating vagal afferents to medullary cardiovascular centers, affecting HR and BP control [4-6]. In addition, the cardioinhibitory reflex, known as the Bezold-Jarisch reflex (BJR), triggers bradycardia and vasodilation. In the reflex, venous return is reduced by elevated intra-abdominal pressure, resulting in insufficient ventricular filling. The activation of ventricular mechanoreceptors paradoxically decreases HR and BP [7]. In some case reports, marked gastrointestinal dilatation caused bradycardia or sinus arrest through these mechanisms [8-10].

**Figure 3:**
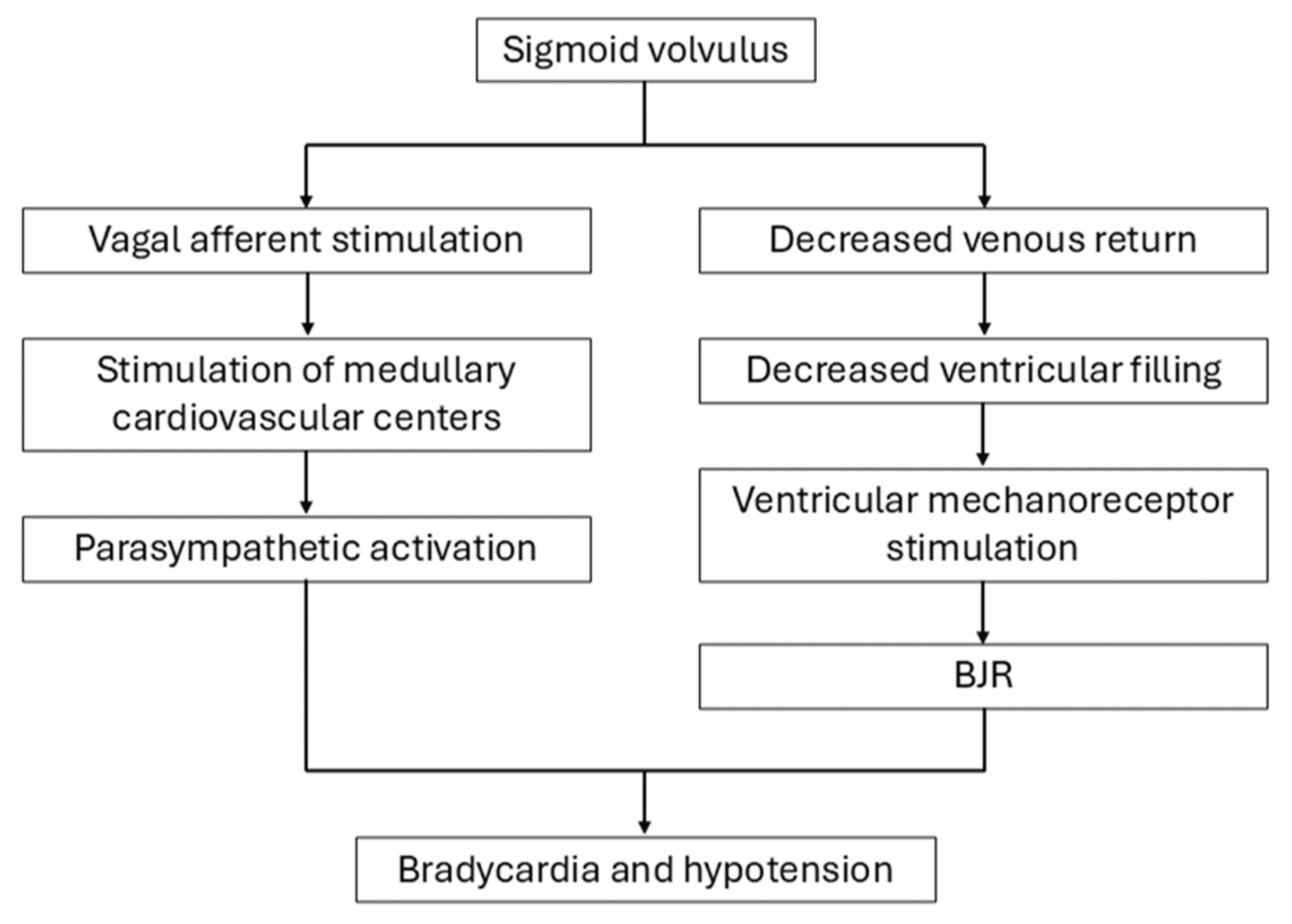
Mechanism of bradycardia and hypotension in sigmoid volvulus Sigmoid volvulus causes marked colonic distension and elevated intra-abdominal pressure, which stimulate vagal afferents to the medullary cardiovascular centers, enhancing parasympathetic tone and resulting in bradycardia. Concurrently, increased intra-abdominal pressure reduces venous return and ventricular filling, activating ventricular mechanoreceptors and the Bezold–Jarisch reflex (BJR). The combined effect of these pathways leads to bradycardia, vasodilation, and ultimately hypotension.

In the ED, major causes of bradycardia and hypotension are cardiogenic, such as arrhythmic, metabolic, electrolyte, or pharmacologic abnormalities [1,2]. However, gastrointestinal diseases are rarely considered in the diagnostic process [3]. To the best of our knowledge, no prior reports clearly demonstrated sigmoid volvulus a direct cause of persistent bradycardia and hypotension. In our case, the patient's hemodynamic resolution following decompression suggests that severe sigmoid dilatation and increased intra-abdominal pressure likely triggered vagal nerve stimulation and BJR, resulting in bradycardia and hypotension.

The bisoprolol may have caused his bradycardia this time. However, given that his vital signs improved immediately after the detorsion, the most likely cause was the effect of sigmoid volvulus. In this case, there were no abdominal symptoms, and the findings were not typical of sigmoid volvulus.

Since this was his first episode of sigmoid volvulus, surgery was not performed. However, surgical treatment will likely be considered if the condition recurs in the future.

## Conclusions

When confronted with unexplained bradycardia and hypotension, clinicians should broaden the differential diagnosis beyond common cardiogenic, metabolic, and pharmacologic etiologies to include gastrointestinal disorders, such as sigmoid volvulus. As demonstrated in this case, even in the absence of typical abdominal symptoms, marked colonic distension can exert significant hemodynamic effects through vagal stimulation and the BJR.

Failure to recognize this potential mechanism may delay appropriate diagnosis and treatment, especially in emergency settings where rapid decision-making is essential. Physicians should promptly stabilize vital signs and proceed with appropriate imaging studies and definitive treatment.

## References

[1] Okamoto A, Someno T, Okada M, Suzuki S (2025). Severe bradycardia and shock with delayed onset due to lacosamide overdose: a case report. Cureus.

[2] Rae AP, Hutton I (1986). Cardiogenic shock and the haemodynamic effects of arrhythmias. Br J Anaesth.

[3] Banerjee A (10.1007/978-3-319-50718-7_1). Cardiovascular emergencies. Emergency Clinical Diagnosis.

[4] Browning KN, Travagli RA (2014). Central nervous system control of gastrointestinal motility and secretion and modulation of gastrointestinal functions. Compr Physiol.

[5] Li WM, Suzuki A (2006). Reflex inhibition of heart rate and efferent cardiac sympathetic outflow induced by colorectal distension in anesthetized rats. J Physiol Sci.

[6] Li WM, Suzuki A, Cui KM (2006). Responses of blood pressure and renal sympathetic nerve activity to colorectal distension in anesthetized rats. J Physiol Sci.

[7] Mark AL (1983). The Bezold-Jarisch reflex revisited: clinical implications of inhibitory reflexes originating in the heart. J Am Coll Cardiol.

[8] Neri E, Caramella D, Vannozzi F, Turini F, Cerri F, Bartolozzi C (2007). Vasovagal reactions in CT colonography. Abdom Imaging.

[9] Sharma G, Boopathy Senguttuvan N, Juneja R, Kumar Bahl V (2012). Neurocardiogenic syncope during a routine colonoscopy: an uncommon malignant presentation. Intern Med.

[10] Stringer R, Schrader M (2017). Acute idiopathic gastric distension causing atrioventricular block and cardiogenic shock. J Emerg Med.

